# Durvalumab after Chemoradiotherapy for PD-L1 Expressing Inoperable Stage III NSCLC Leads to Significant Improvement of Local-Regional Control and Overall Survival in the Real-World Setting

**DOI:** 10.3390/cancers13071613

**Published:** 2021-03-31

**Authors:** Julian Taugner, Lukas Käsmann, Chukwuka Eze, Amanda Tufman, Niels Reinmuth, Thomas Duell, Claus Belka, Farkhad Manapov

**Affiliations:** 1 Department of Radiation Oncology, University Hospital, LMU Munich, 81377 Munich, Germany; Julian.Taugner@med.uni-muenchen.de (J.T.); Chukwuka.Eze@med.uni-muenchen.de (C.E.); Claus.Belka@med.uni-muenchen.de (C.B.); Farkhad.Manapov@med.uni-muenchen.de (F.M.); 2 Comprehensive Pneumology Center Munich (CPC-M), Member of the German Center for Lung Research (DZL), Center for Lung Research (DZL), 81377 Munich, Germany; 3 German Cancer Consortium (DKTK), Partner Site Munich, 80336 Munich, Germany; 4 Division of Respiratory Medicine and Thoracic Oncology, Department of Internal Medicine V, Thoracic Oncology Centre Munich, LMU Munich, 81377 Munich, Germany; Amanda.Tufmann@med.uni-muenchen.de; 5 Asklepios Kliniken GmbH, Asklepios Fachkliniken Muenchen, 82131 Gauting, Germany; n.reinmuth@asklepios.com (N.R.); T.Duell@asklepios.com (T.D.)

**Keywords:** NSCLC, multimodal treatment, stage III, real world data, durvalumab

## Abstract

**Simple Summary:**

Concurrent platinbased chemoradiotherapy followed by maintenance treatment with the PD-L1 inhibitor durvalumab is the new standard of care for inoperable stage III NSCLC. The present study compares the oncological outcome of patients treated with chemoradiotherapy to those treated with chemoradiotherapy and durvalumab (CRT-IO) in the real-world setting. Median follow-up for entire cohort was 33.1 months and median overall survival was 27.2 months. In the CRT-IO cohort after a median follow-up of 20.9 (range: 6.3–27.4) months, local-regional-progression-free-survival, progression-free, and overall survival (PFS, OS) were significantly improved compared to the historical cohort of conventional chemoradiotherapy patients. This real-world analysis demonstrated that durvalumab after chemoradiotherapy (CRT) led to significant improvement of local-regional control, PFS, and OS in PD-L1 expressing inoperable stage III NSCLC patients compared to a historical cohort.

**Abstract:**

Concurrent chemoradiotherapy (CRT) followed by maintenance treatment with the PD-L1 inhibitor durvalumab is a new standard of care for inoperable stage III NSCLC. The present study compares the oncological outcome of patients treated with CRT to those treated with CRT and durvalumab (CRT-IO) in the real-world setting. The analysis was performed based on the retro- and prospectively collected data of 144 consecutive inoperable stage III NSCLC patients treated between 2011–2020. Local-regional-progression-free-survival (LRPFS—defined as progression in the mediastinum, hilum and/or supraclavicular region at both sites and the involved lung), progression-free survival (PFS), and overall survival (OS) were evaluated from the last day of thoracic radiotherapy (TRT). Median follow-up for the entire cohort was 33.1 months (range: 6.3–111.8) and median overall survival was 27.2 (95% CI: 19.5–34.9) months. In the CRT-IO cohort after a median follow-up of 20.9 (range: 6.3–27.4) months, median PFS was not reached, LRPFS (*p* = 0.002), PFS (*p* = 0.018), and OS (*p* = 0.005) were significantly improved vs. the historical cohort of conventional CRT patients. After propensity-score matching (PSM) analysis with age, gender, histology, tumor volume, and treatment mode, and exact matching for T-and N-stage, 22 CRT-IO patients were matched 1:2 to 44 CRT patients. Twelve-month LRPFS, PFS, and OS rates in the CRT-IO vs. CRT cohort were 78.9 vs. 45.5% (*p* = 0.002), 60.0 vs. 31.8% (*p* = 0.007), and 100 vs. 70.5% (*p* = 0.003), respectively. This real-world analysis demonstrated that durvalumab after CRT led to significant improvement of local-regional control, PFS, and OS in PD-L1 expressing inoperable stage III NSCLC patients compared to a historical cohort.

## 1. Introduction

Inoperable stage III non-small-cell lung carcinoma (NSCLC) represents a complex and heterogeneous disease with significant differences regarding patient, tumor, and treatment characteristics [1,2,3,4,5,6,7,8]. In the real-world setting, there is also significant variations in patient prognosis with median survival ranging from 15–30 months and five-year survival rates from 15–32% [9,10,11,12,13]. Continuously during the last decades, chemotherapy and conventionally fractionated thoracic radiotherapy (TRT) represented the standard of care. Several phase III trials including RTOG 73-01, CALGB 8433, RTOG 9410, and RTOG 0617 established platinum-based concurrent chemoradiotherapy (CRT) to a cumulative dose of 60 Gy without induction and consolidation chemotherapy as the most effective strategy accompanied by a moderate acute toxicity profile [14,15,16,17,18]. This treatment paradigm was modified after the pivotal phase III PACIFIC trial showing an unprecedented improvement of progression-free survival (PFS) and overall survival (OS) after consolidation therapy with the Programmed death-ligand 1 (PD-L1) inhibitor durvalumab following platinum-based CRT [19,20,21]. Importantly, histology and molecular tumor profile, including tumor cell PD-L1 expression were not primary stratification factors in this trial. PACIFIC demonstrated very robust PFS improvement across all patient subgroups and led to a rapid implementation of this novel tri-modal approach overseas. However, domestically, based on the results of a post-hoc analysis, the European Medicines Agency (EMA) approved durvalumab consolidation only for PD-L1 positive tumors (≥1%) on initial biopsy.

After PACIFIC, several randomized trials were initiated to confirm and further optimize this novel tri-modal treatment for inoperable Union for International Cancer Control (UICC) stage III NSCLC [22]. In spite of the swift translation of this new standard into the clinical practice, there are still limited data reporting on efficacy of durvalumab consolidation in the real-world setting. Most studies were devoted to the evaluation of patient eligibility for maintenance treatment [23,24,25] or estimation of the risk of pneumonitis after durvalumab [26,27,28]. Hitherto, to the best of our knowledge, there are only a few published reports evaluating its efficacy in a real-life patient cohort. Offin et al. with 62, Jung et al. with 21, and Chu et al. with 31 patients reported promising survival outcomes combined with a moderate increase in pneumonitis [29,30,31].

The purpose of the present study was to analyze oncological outcome of PD-L1 expressing inoperable stage III NSCLC patients and compare it with a historical cohort treated with CRT alone.

## 2. Patients and Methods

### 2.1. Patient Characterstics

This study included 144 consecutive patients who received concurrent or sequential conventionally fractionated CRT with or without consolidation durvalumab as part of the multimodal approach for UICC 8th edition stage IIIA/B/C NSCLC between 2011 and 2020. All 22 patients treated with durvalumab were enrolled starting October 2018 after the European Medicines Agency (EMA) approval. Patients treated without durvalumab either had PD-L1 <1% or were treated prior to durvalumab approval. Patients with follow-up <6 months were excluded. All prospectively enrolled patients gave their informed consent for the use of their data for research purposes. Furthermore, the local ethics committee granted approval to conduct this study (17-230).

All patients were treated at a single tertiary cancer center. Prior to the actual treatment, basic patient characteristics such as tobacco consumption, Eastern Cooperative Oncology Group (ECOG) performance status, and comorbidities, were assessed. As part of the pre-treatment work-up, radiographic imaging was performed using computed tomography (CT) for 9 (6%) patients and positron emission tomography (PET)-CT in 135 (94%) patients. Cranial contrast-enhanced magnetic resonance imaging (MRI) was performed in 79 (55%) patients, while the others received a contrast-enhanced head computed tomography scan (CT). In addition, all patients received routine blood work to assess kidney function as well as complete blood count (CBC) and underwent pulmonary function testing. Patients in the CRT-IO cohort were given durvalumab intravenously at a dose of 10 mg/kg every two weeks up to 12 months (24 cycles), until progression or unacceptable toxicity according to the Common Toxicity Criteria for Adverse Events (CTCAE) version 5. During the course of treatment and prior to application of durvalumab, complete blood work was performed. In addition, pulmonary function tests were performed routinely every 3 months.

All patients were discussed prior to treatment at the multidisciplinary tumor board and all patients were deemed inoperable by an experienced group of thoracic surgeons, pulmonologists, and radiation oncologists. Patients with an initial performance status ECOG > 1, poor lung function (DLCO < 40%, FEV1 < 1l or on long-term oxygen therapy), total RT dose < 60 Gy were excluded from this analysis.

### 2.2. Chemoradiotherapy

All patients underwent PET-CT and/or were planning CT in the treatment position; TRT was planned and delivered in supine position with arms positioned overhead in a WingSTEPT^M^ (Innovative Technologie Völp, Innsbruck, Austria). The gross tumor volume (GTV) and clinical target volume (CTV) were defined according to an in-house standard operating procedure (SOP) in close accordance to the later published ESTRO ACROP guidelines [32]. If patients received induction chemotherapy, only the residual primary tumor volume was contoured, but initially involved lymph-node stations were included in the planning target volume (PTV). To generate the PTV, a margin of 6 mm (axial) and 9 mm (cranio-caudal) added to the CTV.

Conventionally fractionated TRT was administered to the primary tumor and the involved lymph node to a median total dose of 66 Gy. Radiation was delivered on a linear accelerator (LINAC) with a megavoltage capability of (6–15 MV) using 3D-CRT in 48 (33%) patients and intensity-modulated radiotherapy (IMRT) or volumetric modulated arc therapy (VMAT) in 95 (66%) patients. Inter-fraction motion was routinely assessed on cone-beam CT.

### 2.3. Patient Follow-Up

CT/PET-CT scans, routine blood sample, pulmonary function testing, and clinical examinations were performed every 3 months for the first two years after radiotherapy, thereafter twice annually for up to five years, according to an in-house aftercare protocol. Contrast-enhanced brain MRI, bone-scintigraphy, and bronchoscopy were only performed if clinically indicated.

Local-regional recurrence (LRR)—defined as progression in the mediastinum, hilum, and/or supraclavicular region at both sites and the involved lung—along with new distant metastases (DM) and brain metastasis (BM) were documented with CT, PET-CT, and MRI scans. Histological or cytological confirmation of progressive disease was not obligatory. Median follow-up was calculated as the time from the last day of TRT to last follow-up or loss of follow-up.

Progression-free survival (PFS) was defined as the time from end of TRT until disease progression or death. Similarly, overall survival (OS), local-regional-progression-free-survival (LRPFS), and time-to-LRR (TLRR) were assessed from the end of TRT.

### 2.4. Statistical Analysis

To evaluate impact of the addition of durvalumab to routine treatment, OS, PFS, and LRPS were evaluated as primary endpoints. Initially, a univariate analysis of durvalumab treatment and other factors OS, PFS, and LRPFS was conducted in the entire cohort. Multivariate analysis ensued for PFS with other common covariates using Cox regression. Thereafter, we applied Propensity Score Matching (PSM) to reduce confounding using the R plug-in for IBM SPSS 26 [33,34,35,36,37]. The impact of durvalumab treatment was then re-assessed in the matched cohort with univariate analysis using Log-rank testing. *p* < 0.050 were considered significant and *p* < 0.100 a trend. All statistics were performed using IBM SPSS version 26 (IBM, Armonk, NY, USA).

## 3. Results

### 3.1. Patient and Tumor Characteristics

A summary of patient and tumor characteristics of the entire cohort, as well as the CRT-IO and CRT-alone subgroup is shown in Table 1. The entire cohort consisted of 144 consecutive NSCLC patients with inoperable stage IIIA-C (UICC 8th edition) NSCLC treated before and after the durvalumab approval. Patients, which were originally classified according to UICC 7th, were re-grouped according to UICC 8th edition. All patients received conventionally fractionated TRT. Median age was 68.4 with 93 (64.6%) patients older than 65 years. Forty-seven (32.6%) were female and 97 (67.4%) male. On pre-treatment staging, 16 (11.1%), 29 (20.1%), 37 (25.7%), 62 (43.1%) had T1, T2, T3, and T4 disease and 20 (13.9%) N0, 12 (8.3%) N1, 53 (36.8%) N2, and 59 (41.0%) N3 disease, respectively. Fifty (34.7%) patients had UICC stage IIIA, 56 (38.9%) patients IIIB and 38 (26.4%) patients IIIC. Median PTV was 720.0 cc (range: 181–1958). In the histological evaluation, 66 (45.8%) patients had squamous-cell-carcinoma (SCC), 65 (45.1%) had adenocarcinoma (AC) and in 13 (9.0%) of the patients the tumor was classified as NOS. All patients received radiotherapy to a total dose ≥60 Gy (median total dose: 66 Gy, range 60–70 Gy). Concurrent CRT was performed in 122 (84.7%) of patients and 12 (8.3%) patients received sequential chemotherapy and TRT. Ten (7.5%) patients were treated with TRT alone. The predominant concurrent chemotherapy regimen administered in 99 (68.8%) patients consisted of cisplatin given intravenously at a dose of 20 mg/m² on days 1–4 and oral vinorelbine (Navelbine) 50 mg/m² on days 1, 8, and 15, every 4 weeks for two courses according to GILT study [38]. After CRT, 22 (15.3%) patients with tumor PD-L1 ≥ 1% as assessed per VENTANA PD-L1 (SP263) Assay (Roche Diagnostics, F. Hoffmann-La Roche Ltd., Basel, Switzerland), received consolidation durvalumab 10 mg/kg every two weeks for up to 24 cycles (median 14, minimum 2). Median time to the first cycle of durvalumab after the end of CRT was 23 days (range: 13–64). Median duration of durvalumab treatment reached 6.7 months (range: 0.5–12.2). The median follow-up for the entire cohort was 33.1 months (range: 6.3–111.8) median PFS and OS was 9.2 (95% CI: 6.7–11.6) months and 27.2 (95% CI: 19.5–34.9) months, respectively.

### 3.2. Comparison of CRT and CRT-IO in the Entire Cohort

**Figure 1:**
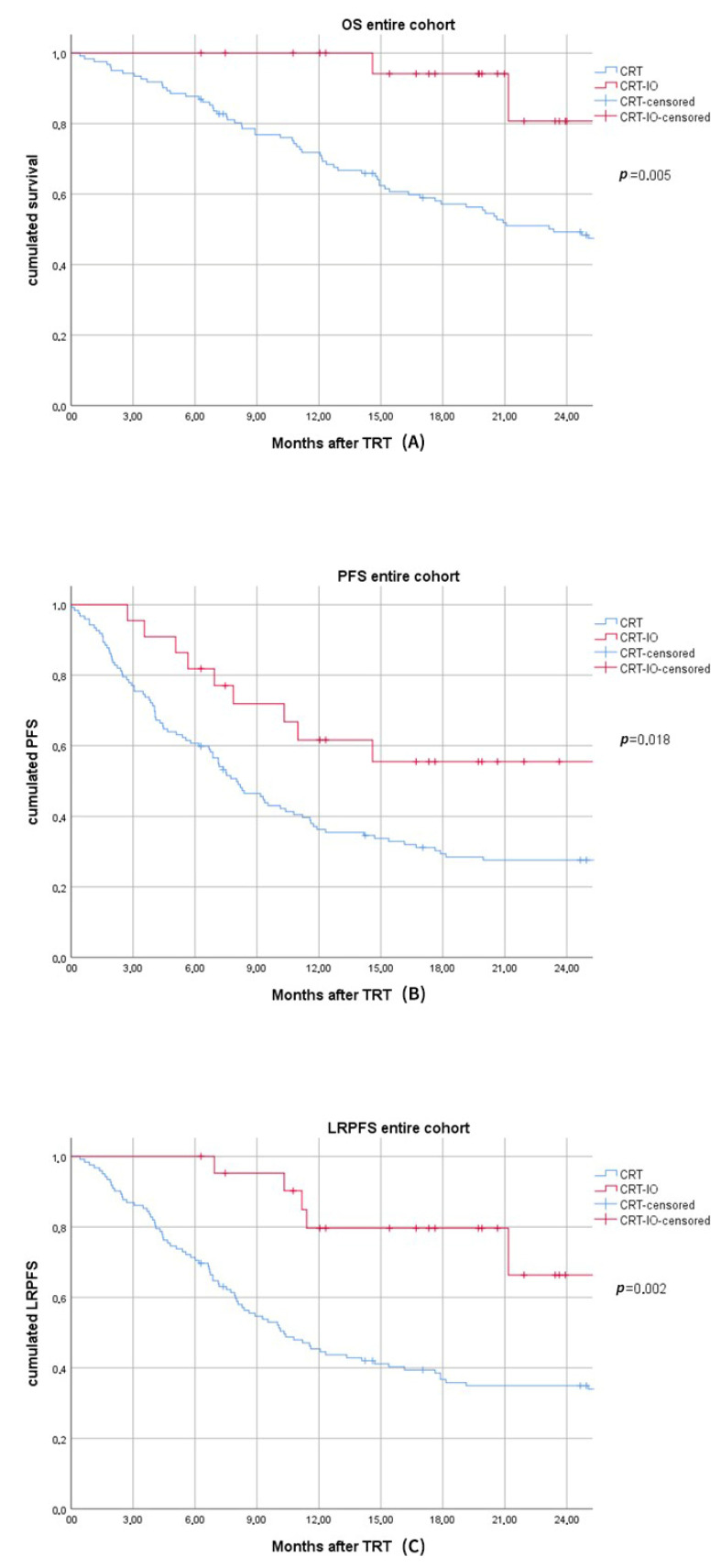
(**A**). Kaplan–Meier curve for overall survival (OS) of chemoradiotherapy (CRT) versus chemoradiotherapy followed by durvalumab maintenance treatment (CRT-IO). (**B**). Kaplan–Meier curve for progression-free survival (PFS) of chemoradiotherapy (CRT) versus chemoradiotherapy followed by durvalumab maintenance treatment (CRT-IO). (**C**). Kaplan–Meier curve for local-regional-progression-free-survival (LRPFS) of chemoradiotherapy (CRT) versus chemoradiotherapy followed by durvalumab maintenance treatment (CRT-IO).

Median follow-up of durvalumab patients reached 19.8 (range: 6.3–27.4) months. Two of 22 durvalumab patients died (14.5 and 21.1 months after end TRT). Median OS of the CRT cohort was 23.4 (95%CI: 17.2–29.6) months. Six-,12- and 18-month OS rates were 100, 100, and 91.6% vs. 87.7, 71.4, and 56.0% in the CRT-IO vs. CRT cohort, respectively (*p* = 0.005) (Figure 1.)

Other significant negative factors for OS in univariate analysis were PTV ≥ 700cc (*p* = 0.045) and histology non-AC (*p* = 0.023). Age > 65 years (*p* = 0.054), male gender (*p* = 0.053) showed a trend and absence of simultaneous chemotherapy had no significant impact on OS (*p* = 0.356).

Median PFS was not reached vs. 8.0 (95% CI: 6.2–9.9) months (*p* = 0.018) in the CRT-IO vs. CRT cohort and the 6-, 12-, and 18-month PFS-rates were 81.8, 60, and 43.8% vs. 60.7, 35.8, and 28.0% respectively (Figure 1.) Age > 65 years (*p* = 0.783), gender (*p* = 0.485), and histology (*p* = 0.972) showed no significant impact on PFS. PTV ≥ 700cc showed a trend as a negative prognosticator for PFS in the entire cohort (*p* = 0.061) as well as N3-situation prior to treatment (*p* = 0.083). Sequence of chemotherapy had no significant impact on PFS (*p* = 0.677). Median LRPFS was not reached in the CRT-IO cohort and 10.3 (95% CI: 7.7–13.0) months in the CRT cohort (*p* = 0.002). LRPFS-rates were 100 and 78.9% vs. 71.3 and 44.5% after 6 and 12 months in the CRT-IO vs. CRT cohort, respectively. After 6 and 12 months 0 and 21.1% vs. 22.8 and 47.1% showed local-regional progress in the CRT-IO and CRT cohort (*p* = 0.002) [Figure 1]. Apart from IO-treatment, only PTV ≥ 700cc (*p* = 0.039) and histology non-AC (*p*= 0.018) had a significant negative impact on LRPFS. After 6 months brain metastasis and other distant metastasis rates were 0 and 18.1% vs. 8.1 and 17.0% in the CRT-IO vs. CRT cohort.

Results of univariate analysis in the entire cohort are displayed in Table 2.

Multivariate analysis was only conducted for PFS, because of the lag of events for OS in the CRT-IO cohort. Variables showing a trend (PTV ≥ 700cc, *p* = 0.061 and N3, *p* = 0.081) or significant impact (IO-treatment, *p* = 0.018) on PFS in univariate analysis were included in the Cox-regression:

For patients treated without CPI the hazard ratio (HR) was 2.072 (95% CI:1.036–4.144, *p* = 0.039). For patients with PTV ≥ 700cc the hazard ratio (HR) was 1.315 (95% CI:0.886–1.951, *p* = 0.174). For patients with initial N3 the hazard ratio (HR) was 1.196 (95% CI:0.806–1.774, *p* = 0.374).

### 3.3. PSM with Exact T- and N-Stage Matching

Patients treated with CPI were matched in a 1:2 ratio to patients treated with CRT alone. Patients treated without concurrent chemotherapy were excluded. To each CRT-IO patient, two corresponding patients with exactly the same T-and N-stage were matched. Twenty-two CRT-IO patients were matched to 44 CRT patients. Both subgroups had patients with matched T- and N-stage. In the CRT-IO subgroup, there were 13 (59.1%) patients aged ≥65, 16 (72.7%) males, 11 (50.0%) with SCC or NOS, and 10 (45.5%) patients with PTV ≥ 700cc. In the CRT subgroup there were 27 (61.4%) patients aged ≥65, 27 (61.4%) males, 22 (50.0%) with SCC or NOS and 17 (38.6) patients with PTV ≥ 700cc. A summary of patient and tumor characteristics is shown in Table 3.

**Figure 2:**
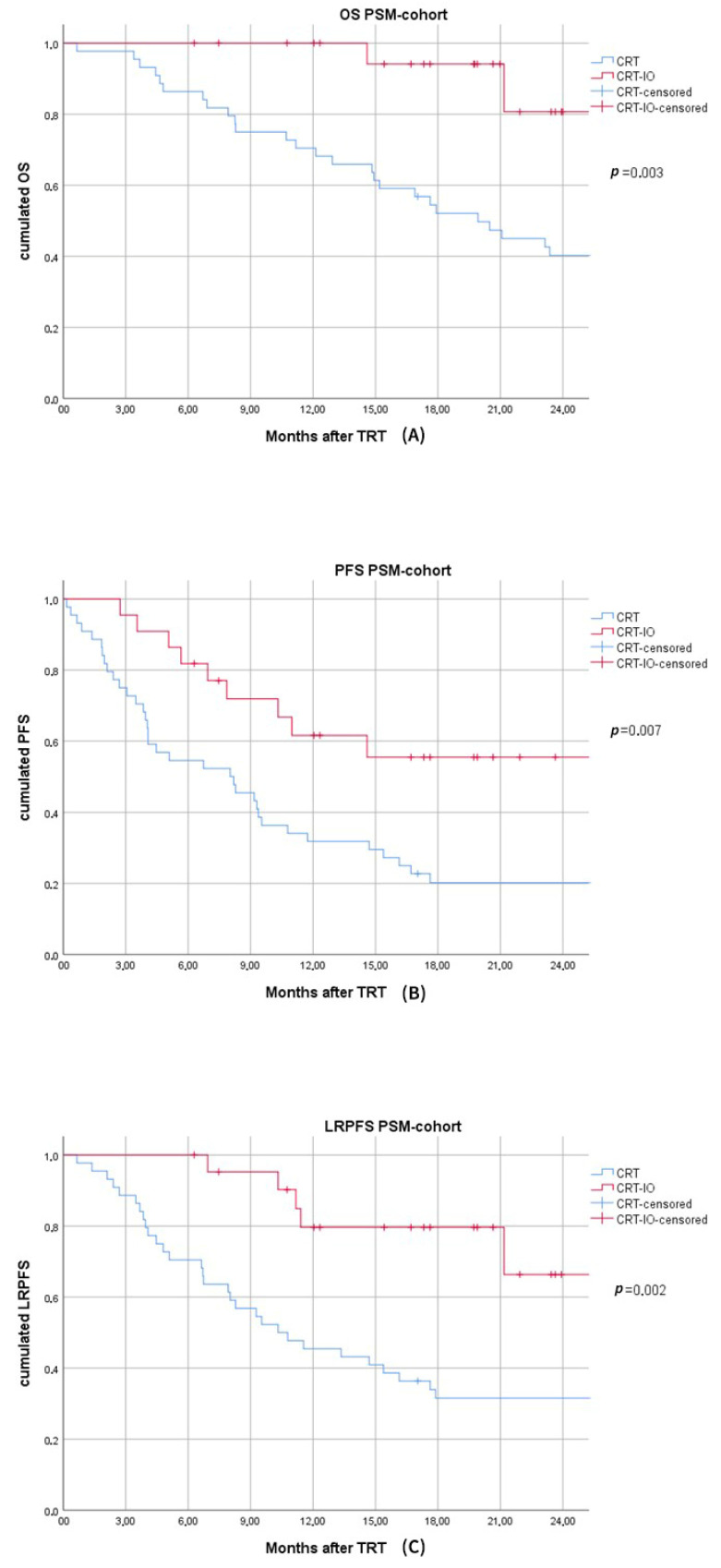
(**A**). Kaplan–Meier curve of the PSM-subgroups for overall survival (OS) of chemoradiotherapy (CRT) versus chemoradiotherapy followed by durvalumab maintenance treatment (CRT-IO). (**B**). Kaplan–Meier curve of the PSM-subgroups for progression-free survival (PFS) of chemoradiotherapy (CRT) versus chemoradiotherapy followed by durvalumab maintenance treatment (CRT-IO). (**C**). Kaplan–Meier curve of the PSM-subgroups for local-regional-progression-free-survival (LRPFS) of chemoradiotherapy (CRT) versus chemoradiotherapy followed by durvalumab maintenance treatment (CRT-IO).

The median follow-up of the PSM-cohort was 27.4 months (range: 6.3–111.8); median PFS and OS was 9.4 (95% CI: 6.5–12.2) and 27.2 (95% CI: 14.9–39.5) months, respectively; 6- and 12-month OS-rates were 100 and 100% vs. 86.4 and 70.5% in the CRT-IO vs. CRT-cohort, respectively (*p* = 0.003) [Figure 2]. After 6 and 12 months, 81.8 and 60.0% vs. 54.5 and 31.8% of CRT-IO vs. CRT patients were alive and without progression (*p* = 0.007) [Figure 2]. LRPFS 6 and 12 months after TRT was 100 and 78.9% in the CRT-IO vs. 70.5 and 45.5% in the CRT subgroup (*p* = 0.002) (Figure 2).

After 6 months, rates of brain metastasis and other distant metastasis rates were 0 and 18.1% vs. 7.7 and 26.2% in the CRT-IO vs. CRT cohort.

## 4. Discussion

The aim of this study was to analyze the oncological outcome of PD-L1 expressing inoperable stage III NSCLC patients treated with maintenance durvalumab after CRT and to compare it with an otherwise similarly treated historical cohort. Additionally, PSM analysis with matching for principal patient-, tumor-, and treatment characteristics was conducted to account for bias.

The present study revealed a significant improvement across all survival parameters in the CRT-IO versus CRT patients. The most pronounced difference was found for OS. Estimated 6- and 12- month survival rates were 100 vs. 87.7% and 100 vs. 72.1% in the CRT-IO and CRT cohort, respectively. Vis-à-vis PFS, continuously higher rates at 6-, 12-, and 18-months after the end of TRT were achieved in patients treated with versus without durvalumab. Another important finding was a remarkable increase in the local-regional control rates across all tested time points. These results were confirmed after PSM analysis. Comparing the oncological outcome in the 66 matched patients substantial increase in all survival parameters was again revealed.

Hence, the present analysis corroborated PACIFIC findings in the real-world setting. Furthermore, 50%/14% of CRT-IO patients in our study presented with UICC stage IIIB/IIIC disease with a median PTV of 680.3 ccm, thus representing a high-risk cohort. Nevertheless, all started with durvalumab maintenance after completion of CRT and demonstrated promising results especially for the local-regional control.

The role of local-regional control after CRT for patient survival was described. Machtay et al. analyzed 1390 patients treated with CRT within the scope of seven RTOG trials and found a highly significant association of local-regional control and OS [39]. The predicted 18-month LRPFS and OS rates in our analysis of patients treated with CTR-IO were very favorable at about 70 and 91.6%, respectively.

The present study was also in close accordance with a study by Offin et al. on 62 inoperable stage III patients treated with CRT-IO at the Memorial Sloan Kettering Cancer Center (29). Other real-life studies from Jung et al. and Chu et al. included 21 and 31 stage III patients treated with durvalumab after CRT and revealed similar PFS rates [30,31]. Comparing these single-center studies to each other, a plateau of the PFS curves starting after completion of durvalumab treatment could be observed. The same trend was also seen in the original analysis of the PACIFIC trial [19,20]. This interesting finding was not described in earlier CRT studies for inoperable stage III NSCLC. A short review of literature of real-world experiences using chemo/radiation followed by consolidative durvalumab is shown in Table 4.

Therefore, we can hypothesize that an immunological phenomenon may be associated with the establishment of a long-lasting anti-tumor response in these patients. However, a clarification of this phenomenon will be solely possible with longitudinal analyses of immunological cellular and humoral factors across all time points before, during and after durvalumab treatment.

In contrast to previously published studies on durvalumab efficacy, it is pertinent to mention that our durvalumab cohort exclusively included patients with PD-L1 expressing tumors at initial biopsy. Nevertheless, we found similar survival rates, which is also a relevant finding. Recently Desilets et al. reported an improved 12-month OS in durvalumab-treated patients with PD-L1 expression ≥50% [40]. OS and PFS findings were in close accordance to our data. In order to elucidate these findings, a comparison of oncological outcome in PD-L1 expressing and non-expressing tumors should be performed in larger prospective cohorts. Concerning the significant improvement of LRPFS observed in our patients treated with durvalumab maintenance, Abe et al. describe a similarly improved local control in patients treated with cCRT and durvalumab vs. with cCRT alone [41]. According to Ohri et al., the neutrophil-to-lymphocyte ratio (NLR) after RT may be an important prognostic factor for patients enrolled in durvalumab maintenance, especially concerning PFS [42].

Notwithstanding, the current analysis had its inherent limitations; the single-center design in a limited number of patients and a relatively short follow up period in the CRT-IO cohort must be mentioned. Nevertheless, the reported survival data were in line with other real-world studies and PACIFIC [19,20,29,30,31]. Furthermore, a comprehensive statistical evaluation including PSM analysis with exact T- and N-stage matching was conducted to confirm our findings. In addition, durvalumab maintenance treatment after CRT in non-operable stage III NSCLC is depending on PD-L1 status in the European Union. As a result, our study enrolled only patients with PD-L1 ≥ 1% at initial biopsy and our findings cannot be translated in patients with PD-L1 < 1%.

In summary, our analysis confirmed a significant and robust improvement of oncological outcome in PD-L1 expressing inoperable stage III NSCLC patients treated with CRT and consolidation durvalumab compared to a historical cohort. This improvement mostly consisted of a striking increase in local-regional control and PFS. This increase subsequently translated into improved overall survival.

## 5. Conclusions

This real-world analysis demonstrates that durvalumab after CRT led to significant improvement of local-regional control, PFS, and OS in PD-L1 expressing inoperable stage III NSCLC patients compared to a historical cohort.

## Figures and Tables

**Table 1:** Patient and tumor characteristics of entire cohort and chemoradiotherapy (CRT) vs. chemoradiotherapy and durvalumab (CRT-IO) and the Propensity Score Matching (PSM) CRT subgroup.

	Entire Cohort N (%)	CRT Subgroup N (%)	PSM-CRT Subgroup N (%)	CRT-IO Subgroup N (%)
**Total**	**144**	**122**	**44**	**22**
**Age**				
median years	68.4	68.5	67.9	67.6
>65 years	93 (64.6)	80 (65.6)	27 (61.4)	13 (59.1)
**Gender**				
Male	97 (67.4)	81 (66.4)	27 (61.4)	16 (72.7)
Female	47 (32.6)	41 (33.6)	17 (38.6)	6 (27.3)
**T-stage**				
1	16 (11.1)	15 (12.3)	2 (4.5)	1 (4.5)
2	29 (20.1)	24 (19.7)	20 (22.7)	5 (22.7)
3	37 (25.7)	30 (24.6)	14 (31.8)	7 (31.8)
4	62 (43.1)	53 (43.4)	18 (40.9)	9 (40.9)
**N-stage**				
0	20 (13.9)	15 (12.3)	8 (18.2)	4 (18.2)
1	12 (8.3)	11 (9.0)	2 (4.5)	1 (4.5)
2	53 (36.8)	42 (34.4)	22 (50.0)	11 (50.0)
3	59 (41.0)	54 (44.3)	12 (27.3)	6 (27.3)
**UICC-stage**				
IIIA	50 (34.7)	42 (34.4)	16 (36.4)	8 (36.4)
IIIB	56 (38.9)	45 (36.9)	22 (50.0)	11 (50.0)
IIIC	38 (26.4)	35 (28.7)	6 (13.6)	3 (13.6)
**Planning target volume (PTV)-size**				
median cc	720.1	732.0	634.9	680.3
≥700 ccm	75 (52.1)	65 (53.3)	17 (38.6)	10 (45.5)
**Histology**				
Squamous cell carcinoma (SCC)	66 (45.8)	57 (46.7)	21 (47.7)	9 (40.9)
Adenocarcinoma (AC)	65 (45.1)	54 (44.3)	22 (50.0)	11 (50.0)
Not otherwise specified (NOS)	13 (9.0)	11 (9.0)	1 (2.3)	2 (9.1)
**Radiographic imaging**				
PET-CT	135 (93.8)	114 (93.4)	41 (93.2)	21 (95.5)
cMRI	79 (54.9)	59 (48.4)	25 (56.8)	20 (90.9)
**Treatment**				
Concurrent chemoradiation (CRT)	122 (84.7)	100 (82.0)	44 (100)	22 (100)
Induction chemotherapy	60 (41.6)	54 (44.3)	21 (47.7)	6 (27.3)
3DCRT	48 (33.3)	48 (39.3)	17 (38.6)	0 (0)
IMRT/VMAT	96 (66.7)	74 (60.7)	27 (61.4)	22 (100)
**Median-FU months**	33.1	49.9	62.0	19.8
**OS**				
6-months	129 (89.6)	107 (87.7)	38 (86.4)	22 (100)
12-months	103 (75.4)	85 (71.4)	31 (70.5)	19 (100)
**Progression-free survival (PFS)**				
6-months	92 (63.8)	74 (60.7)	24 (54.5)	18 (81.8)
12-months	55 (39.3)	43 (35.8)	14 (31.8)	12 (60.0)
**Local-regional-free-survival (LRPFS)**				
6-months	109 (75.7)	87 (71.3)	31 (70.5)	22 (100)
12-months	69 (51.4)	54 (44.5)	20 (45.5)	15 (78.9)

**Table 2:** Results of univariate analysis of the entire cohort (Log-Rank).

	Entire Cohort N (%)	OS *p*	PFS *p*	LRPFS *p*
**Total**	144 (100)			
**Age**				
>65 years	93 (64.6)	0.054	0.783	0.475
**Gender**				
Male	97 (67.4)	0.053	0.485	0.324
**T-stage**				
4	62 (43.1)	0.694	0.757	0.278
**N-stage**				
3	59 (41.0)	0.522	0.083	0.370
**UICC-stage**				
IIIC	38 (26.4)	0.320	0.150	0.108
**PTV-size**				
≥700 ccm	75 (52.1)	0.045	0.061	0.039
**Histology**				
SCC + NOS	79 (54.9)	0.023	0.972	0.018
**Treatment**				
Induction chemotherapy	60 (41.6)	0.269	0.214	0.111
Absence of concurrent chemoradiation	23 (16.0)	0.356	0.699	0.382
3DCRT	48 (33.3)	0.223	0.531	0.374
**Durvalumab consolidation**	22 (15.3)			
CRT-IO		0.005	0.018	0.002

**Table 3:** Comparison of the CRT-IO subgroup and the PS-matched CRT subgroup.

	PSM-CRT Subgroup N (%)	CRT-IO Subgroup N (%)	*p*-Value
**Total**	**44**	**22**	
**Age**			
median years	67.9	67.6	
>65 years	27 (61.4)	13 (59.1)	0.895
**Gender**			
Male	27 (61.4)	16 (72.7)	
Female	17 (38.6)	6 (27.3)	0.246
**T-stage**			
1	2 (4.5)	1 (4.5)	
2	20 (22.7)	5 (22.7)	
3	14 (31.8)	7 (31.8)	
4	18 (40.9)	9 (40.9)	0.078
**N-stage**			
0	8 (18.2)	4 (18.2)	
1	2 (4.5)	1 (4.5)	
2	22 (50.0)	11 (50.0)	
3	12 (27.3)	6 (27.3)	0.468
**UICC-stage**			
IIIA	16 (36.4)	8 (36.4)	
IIIB	22 (50.0)	11 (50.0)	
IIIC	6 (13.6)	3 (13.6)	0.663
**PTV-size**			
median cc	634.9	680.3	
≥700 ccm	17 (38.6)	10 (45.5)	0.608
**Histology**			
Squamous cell carcinoma (SCC)	21 (47.7)	9 (40.9)	
Adenocarcinoma (AC)	22 (50.0)	11 (50.0)	
Not otherwise specified (NOS)	1 (2.3)	2 (9.1)	0.066
**Radiographic imaging**			
PET-CT	41 (93.2)	21 (95.5)	0.977
cMRI	25 (56.8)	20 (90.9)	0.762
**Treatment**			
Concurrent chemoradiation (CRT)	44 (100)	22 (100)	0.303
Induction chemotherapy	21 (47.7)	6 (27.3)	**0.009**
**Median-FU months**	62.0	19.8	
**OS**			
6-months	38 (86.4)	22 (100)	
12-months	31 (70.5)	19 (100)	
**PFS**			
6-months	24 (54.5)	18 (81.8)	
12-months	14 (31.8)	12 (60.0)	
**LRPFS**			
6-months	31 (70.5)	22 (100)	
12-months	20 (45.5)	15 (78.9)	

**Table 4:** Short review of literature of real-world experiences using chemo/radiation followed by consolidative durvalumab.

Authors	Title	Year	Results
Michael Offin et al. [29]	Clinical outcomes, local–regional control, and the role for metastasis-directed therapies in stage III non-small cell lung cancers treated with chemoradiation and durvalumab	2020	62 NSCLC stage III patients treated with CRT+ durvalumab. Median follow-up for all patients was 12 months. Estimated 12-month PFS 65% (95% CI: 51–79%) and OS 85% (95% CI: 75–95%). 12-month incidence of local–regional and distant failures were 18% (95% CI: 5.9–30%) and 30% (95% CI: 16.3–44.5%). High tumor mutation burden or PD-L1 did not predict improved PFS.
Hyun Ae Jung et al. [30]	Real world data of durvalumab consolidation after chemoradiotherapy in stage III non-small-cell lung cancer	2020	21 NSCLC stage III patients treated with CRT+ durvalumab. Median PFS of all patients: not reached versus 9.6 (95 % CI 4.5–14.8) months (*p* = 0.060). Durvalumab consolidation treatment was associated with favorable PFS in patients who did not meet the criteria of the PACIFIC study.
Chia-Hsun Chu et al. [31]	Consolidation treatment of durvalumab after chemoradiation in real-world patients with stage III unresectable non-small cell lung cancer	2020	31 NSCLC stage III patients treated with CRT+ durvalumab. 12-month PFS and time to metastatic disease or death-free rate were 56.4 and 66.9%, respectively. Patients with low neutrophil-to-lymphocyte ratio showed a significantly longer post-CRT PFS (*p* = 0.040).
Nitin Ohri et al. [42]	Who benefits the most from adjuvant durvalumab after chemoradiotherapy for non-small cell lung cancer? An exploratory analysis	2020	35 NSCLC stage III patients treated with CRT+ durvalumab, 70 patients treated with CRT alone. Patients treated with CRT+ durvalumab had significantly improved 12-month-PFS of 67 vs. 39% (*p* = 0.006) and 12-month-OS of 88 vs. 76% (*p* = 0.041). Neutrophil-to-lymphocyte ratio <4.3 after TRT was associated with improved PFS in the durvalumab cohort.
Takanori Abe et al. [41]	Effect of durvalumab on local control after concurrent chemoradiotherapy for locally advanced non-small cell lung cancer in comparison with chemoradiotherapy alone	2020	44 NSCLC stage III patients treated with CRT+ durvalumab, 76 patients treated with CRT alone. Median follow-up 17 months. 12-months local-control, distant metastasis, PFS, and OS rates (from start of TRT) 86, 29, 58, and 84% in the CRT+ durvalumab vs. 62, 31, 57, and 89% in the CRT alone cohort. Local control was significantly improved in the durvalumab cohort (*p* = 0.005).
Antoine Desilets et al. [40]	Durvalumab therapy following chemoradiation compared with a historical cohort treated with chemoradiation alone in patients with stage III non-small cell lung cancer: A real-world multicentre study	2020	147 NSCLC stage III patients treated with CRT+ durvalumab, 121 patients treated with CRT alone. Median OS not reached for CRT + durvalumab vs. 26.9 months in CRT patients (*p* = 0.001). Improved 12-month OS in patients with PD-L1 expression ≥50% in the durvalumab cohort (100% vs. 86%, *p* = 0.007)
Present study	Durvalumab after chemoradiotherapy for PD-L1 expressing inoperable stage III NSCLC leads to significant improvement of the local-regional control and overall survival in the real-world setting	2020	22 NSCLC stage III patients treated with CRT+ durvalumab, 122 patients treated with CRT alone. Median follow-up 19.8 months. After PSM 12-month LRPFS, PFS, and OS-rates in the CRT-IO vs. CRT cohort were 78.9 vs. 45.5% (*p* = 0.002), 60.0 vs. 31.8% (*p* = 0.007) and 100 vs. 86.4% (*p* = 0.003), respectively

## Data Availability

The data presented in this study are available from the corresponding author on reasonable request.
